# Supplementary material for: Targeting KRAS4A splicing through the RBM39/DCAF15 pathway inhibits cancer stem cells
Source: Nat Commun. 2021 Jul 13;12:4288. doi: 10.1038/s41467-021-24498-7 (PMC8277813; doi:10.1038/s41467-021-24498-7)
Supplement: Supplementary file 1 — Supplementary Information [file 41467_2021_24498_MOESM1_ESM.pdf]

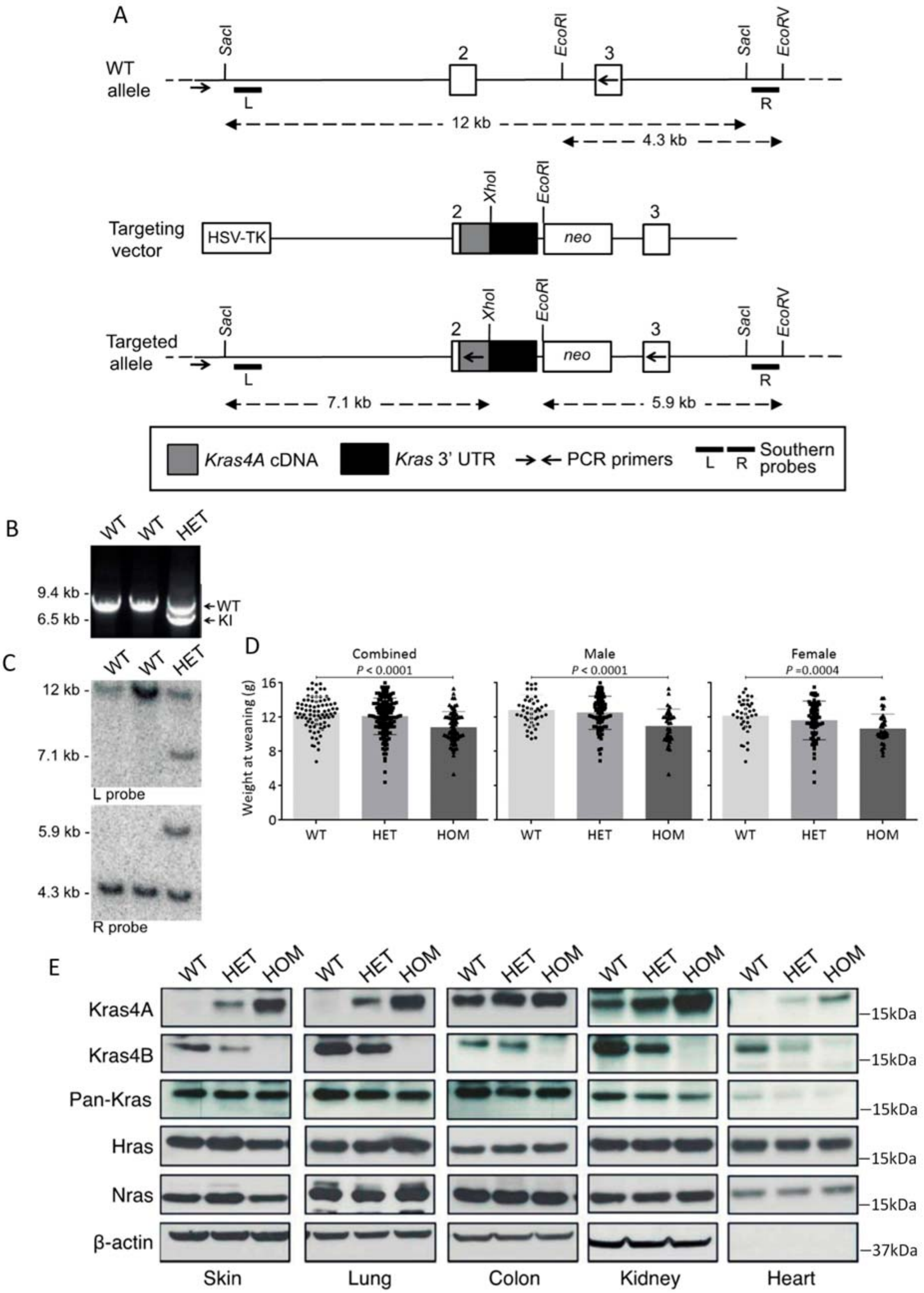

A
WT allele
SacI
2
EcoRI
3
SacI
EcoRV
L
R
12 kb
4.3 kb
Targeting vector
HSV-TK
XhoI
neo
Targeted allele
7.1 kb
5.9 kb
Kras4A cDNA
Kras 3' UTR
PCR primers
Southern probes
B
WT WT HET
9.4 kb
6.5 kb
WT
KI
C
12 kb
7.1 kb
L probe
5.9 kb
4.3 kb
R probe
D
Combined
P < 0.0001
Male
P < 0.0001
Female
P =0.0004
Weight at weaning (g)
16
12
8
4
0
WT HET HOM
E
Kras4A
Kras4B
Pan-Kras
Hras
Nras
β-actin
15kDa
37kDa
Skin
Lung
Colon
Kidney
Heart

Figure S1. Generation and characterization of mice that only express *Kras4A* from the *Kras* locus (*Kras4B-/-*). *(A)* Targeting strategy for the generation of a *Kras* allele with *Kras4A* cDNA knocked into the endogenous locus. *Kras4A* cDNA spanning a portion of exon 2 through exon 4A (in grey) and 3' UTR (in black) was homologously inserted into the *Kras* locus between exons 2 and 3. Locations of restriction endonuclease sites and fragment sizes, PCR screening primers and Southern probes are indicated. *(B-C)* PCR reactions *(B)* and Southern blots *(C)* show the expected DNA fragment sizes for successful targeting of the *Kras4A* allele. *(D)* Weights of WT (n=83), *Kras4B* heterozygous (HET) (n=152) and homozygous *Kras4B-/-* (HOM) (n=70) mice at weaning reveal a mild but significant reduction in weight of homozygous animals. This effect was not gender specific. Data are presented as mean ± s.d. *P* value were calculated by unpaired two-tailed *t*-test. *(E)* Western blot analysis of Kras4A, Kras4B, total Kras (Pan-Kras), Hras, and Nras across five normal tissues reveals expected changes in Kras4A and Kras4B between genotypes, and comparable levels of total Kras, Hras, and Nras across genotypes. β-actin is included as a loading control, although its expression is not detected in heart. Results were consistent across multiple mice from mixed B6/129 as well as pure FVB/N backgrounds. HET and HOM indicate *Kras4B* heterozygous and homozygous knockout genotypes, respectively.

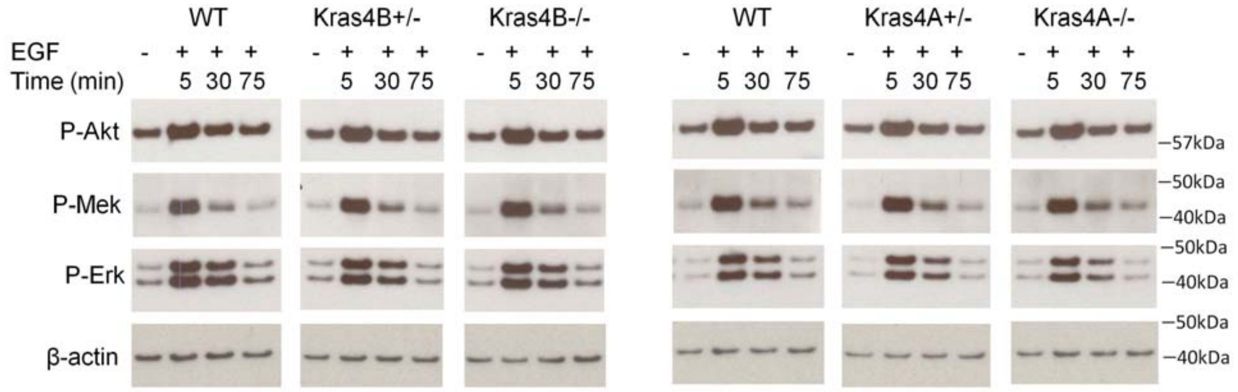

Figure S2. Effect of Kras isoforms on EGF-induced downstream signaling effectors. Analysis of phosphorylation of Akt, Mek and Erk in WT, Kras4A and Kras4B knockout MEFs showed elevated levels by EGF at the 5-minute time point. No apparent differences in EGF-induced activation of Akt, Mek and Erk were observed among mice of the indicated genotypes.

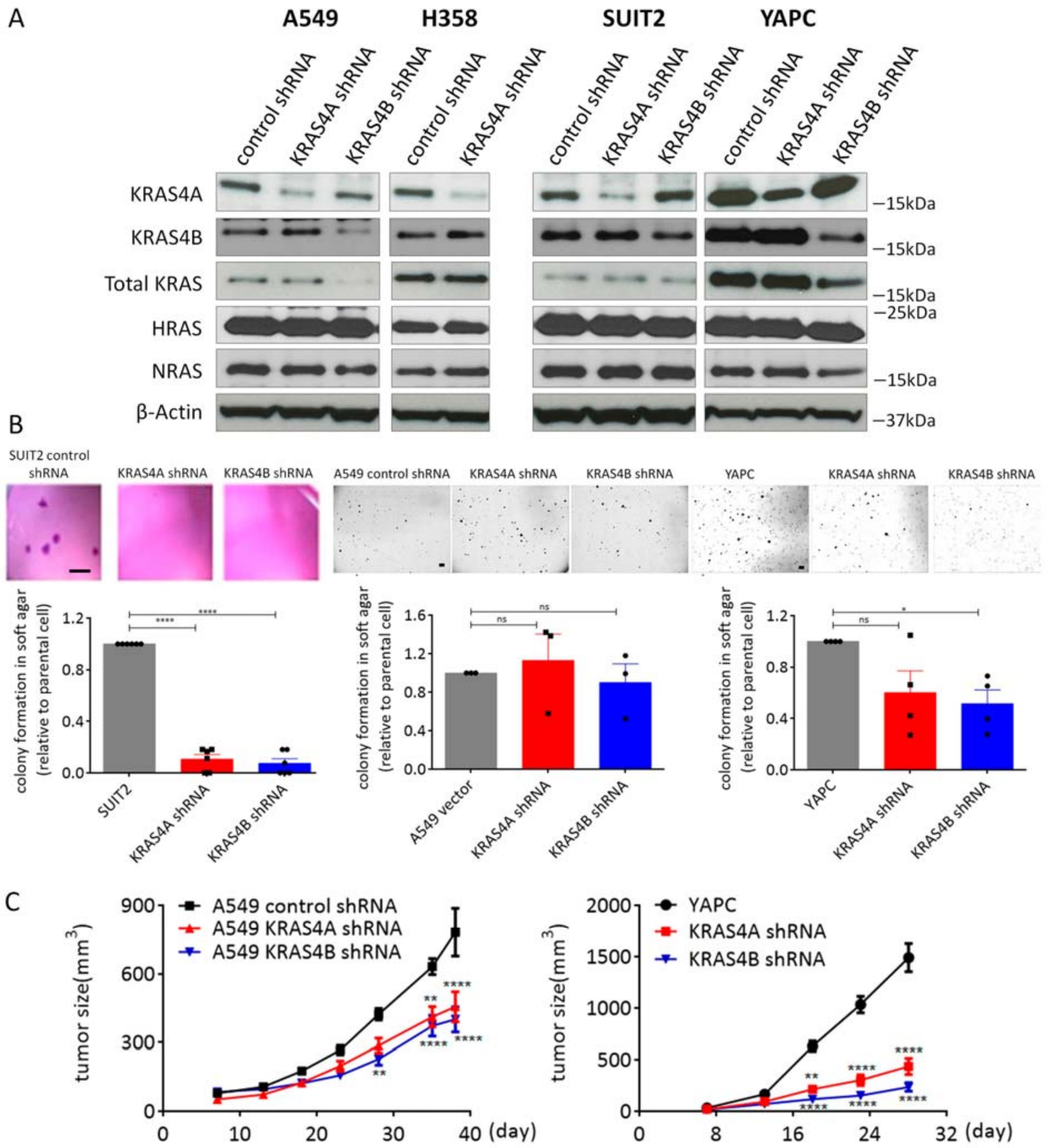

Figure S3. Ras protein levels in KRAS4A and KRAS4B knockdown human cancer cells. Lung cancer cells, A549 and H358, and pancreatic cancer cells, SUIT2 and YAPC, were transduced with indicated targeting shRNA, followed by puromycin selection. (A) RAS levels of the protein extracts from these selected cells with shRNA transfection were determined by western blotting. (B) The growth of SUIT2, A549 and YAPC cells with shRNA were determined by soft agar assay. Data are presented as mean ± s.e.m from n=6 independent experiments in SUIT2 and n=3 independent experiments in A549 and YAPC cells. **P* < 0.05; ****P* < 0.001; *****P* < 0.0001 by one-way ANOVA with Tukey's multiple comparison. The scale bar is presented as 500 µm. (C) Growth of knockdown *KRAS4A* and *KRAS4B* A549 and YAPC cells after subcutaneous injection into immunocompromised mice. Data are presented as mean ± s.e.m. n = 10 mice for

parental YAPC cell and n = 5 mice for parental A549, *KRAS4A* knockdown or *KRAS4B* KD knockdown cell. **$P < 0.01$; ****$P < 0.0001$ by two-way ANOVA with Bonferroni's multiple comparisons test.

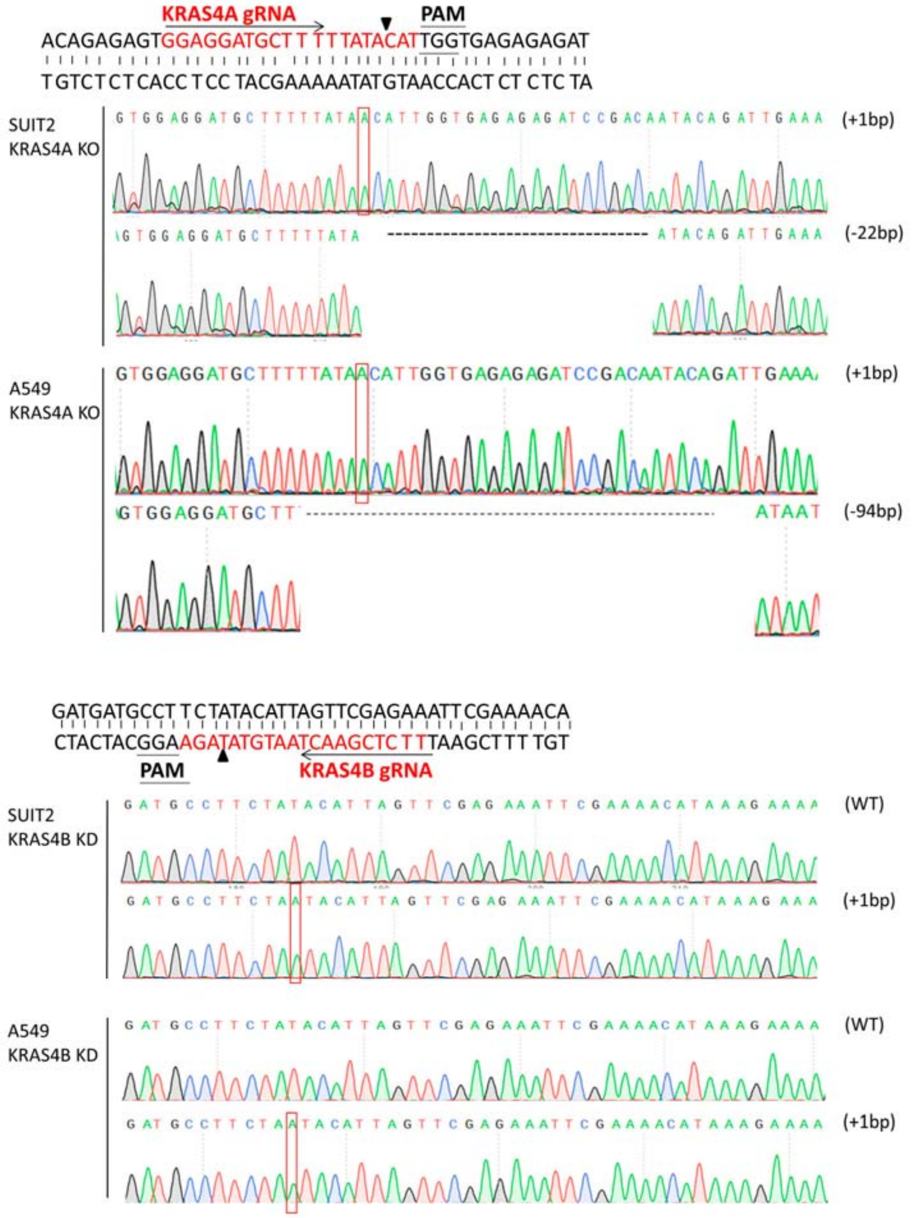

Figure S4. CRISPR/Cas9-based editing of the KRAS4A and KRAS4B exons. The actual indels of each allele in KRAS4A knockout and KRAS4B knockdown cells were determined by sanger sequencing.

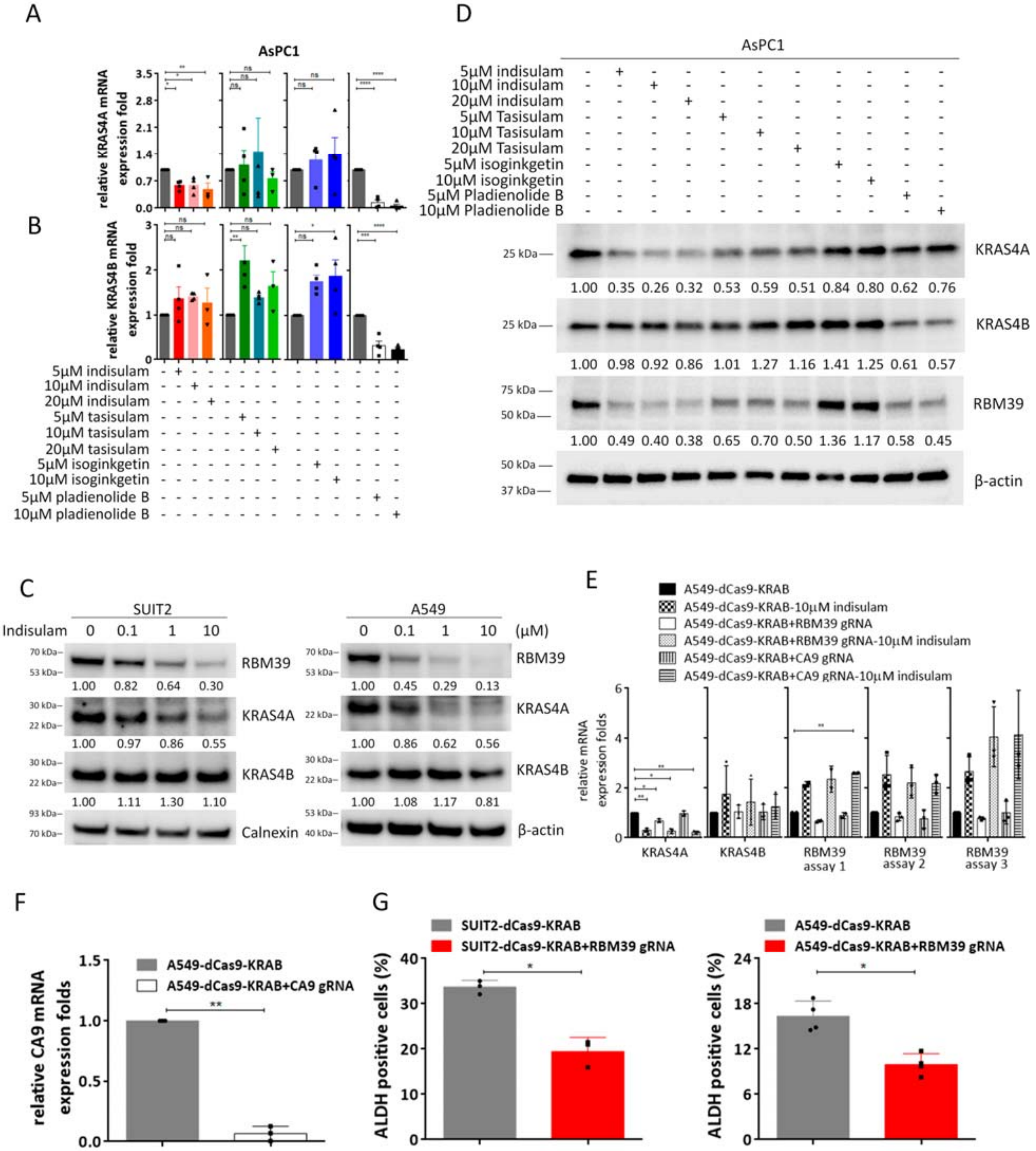
A
B
C
D
E
F
G
AsPC1
relative KRAS4A mRNA expression fold
relative KRAS4B mRNA expression fold
5µM indisulam
10µM indisulam
20µM indisulam
5µM tasisulam
10µM tasisulam
20µM tasisulam
5µM isoginkgetin
10µM isoginkgetin
5µM pladienolide B
10µM pladienolide B
5µM Tasisulam
10µM Tasisulam
20µM Tasisulam
5µM Pladienolide B
10µM Pladienolide B
KRAS4A
1.00 0.35 0.26 0.32 0.53 0.59 0.51 0.84 0.80 0.62 0.76
KRAS4B
1.00 0.98 0.92 0.86 1.01 1.27 1.16 1.41 1.25 0.61 0.57
RBM39
1.00 0.49 0.40 0.38 0.65 0.70 0.50 1.36 1.17 0.58 0.45
β-actin
SUIT2
A549
Indisulam 0 0.1 1 10 (µM)
RBM39
1.00 0.82 0.64 0.30
1.00 0.45 0.29 0.13
KRAS4A
1.00 0.97 0.86 0.55
1.00 0.86 0.62 0.56
KRAS4B
1.00 1.11 1.30 1.10
1.00 1.08 1.17 0.81
Calnexin
β-actin
A549-dCas9-KRAB
A549-dCas9-KRAB-10µM indisulam
A549-dCas9-KRAB+RBM39 gRNA
A549-dCas9-KRAB+RBM39 gRNA-10µM indisulam
A549-dCas9-KRAB+CA9 gRNA
A549-dCas9-KRAB+CA9 gRNA-10µM indisulam
relative mRNA expression folds
KRAS4A
KRAS4B
RBM39 assay 1
RBM39 assay 2
RBM39 assay 3
relative CA9 mRNA expression folds
A549-dCas9-KRAB
A549-dCas9-KRAB+CA9 gRNA
SUIT2-dCas9-KRAB
SUIT2-dCas9-KRAB+RBM39 gRNA
A549-dCas9-KRAB
A549-dCas9-KRAB+RBM39 gRNA
ALDH positive cells (%)

Figure S5 The RBM39 RNA-binding protein mediates *KRAS4A* splicing. The *KRAS4A* (A) and *KRAS4B* (B) mRNA levels were assessed by TaqMan analysis in AsPC1 cells after small molecule inhibitor treatment for 48hr. The specific inhibitors used and their concentrations are shown below the plots. Data are presented as mean ± s.e.m from n=4 independent experiment. **P* < 0.05; ***P* < 0.01; ****P* < 0.001; *****P* < 0.0001 by one-way ANOVA with Dunnetts's multiple comparison. (C) Dose–dependent effects of indisulam on RBM39 and KRAS4A levels. (D) The KRAS4A, KRAS4B and RBM39 protein levels were assessed in AsPC1 by Western blotting after small molecule inhibitor treatment for 48hr. Quantification of KRAS4A, KRAS4B and RBM39 levels was carried out using imageJ software. (E-F) Two sgRNAs targeting *RBM39* or *CA9* were transfected into BFP+ A549 cells stably expressing dCas9-KRAB. Cells were incubated with small molecule inhibitors for 48hr and then analyzed by Taqman analysis. Data are presented as mean ± s.e.m from n=3 independent experiment. **P* < 0.05; ***P* < 0.01 by one-way ANOVA with Dunnetts's multiple comparison. (G) Two sgRNAs targeting *RBM39* were transfected into BFP+ SUIT2 or A549 cells stably expressing dCas9-KRAB. The proportion of *ALDH* expression levels were assessed by fluorescence-activated cell sorting analysis. Data are presented as mean ± s.d from n=3 independent experiments in SUIT2-dCas9-KRAB cells and n=4 independent experiments in A549-dCas9-KRAB cells. **P* < 0.05 by paired two-tailed *t*-test.

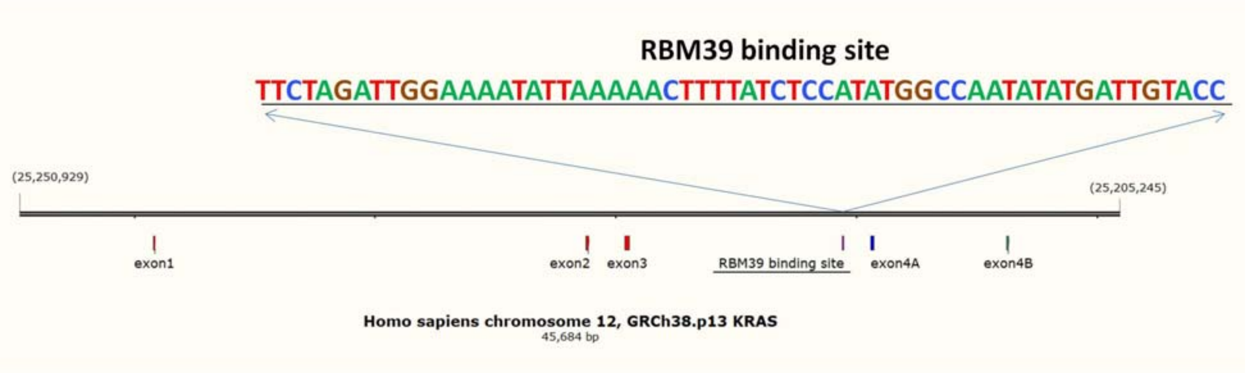

Figure S6. RBM39 PAR-CLIP tags in KRAS near exon 4A.

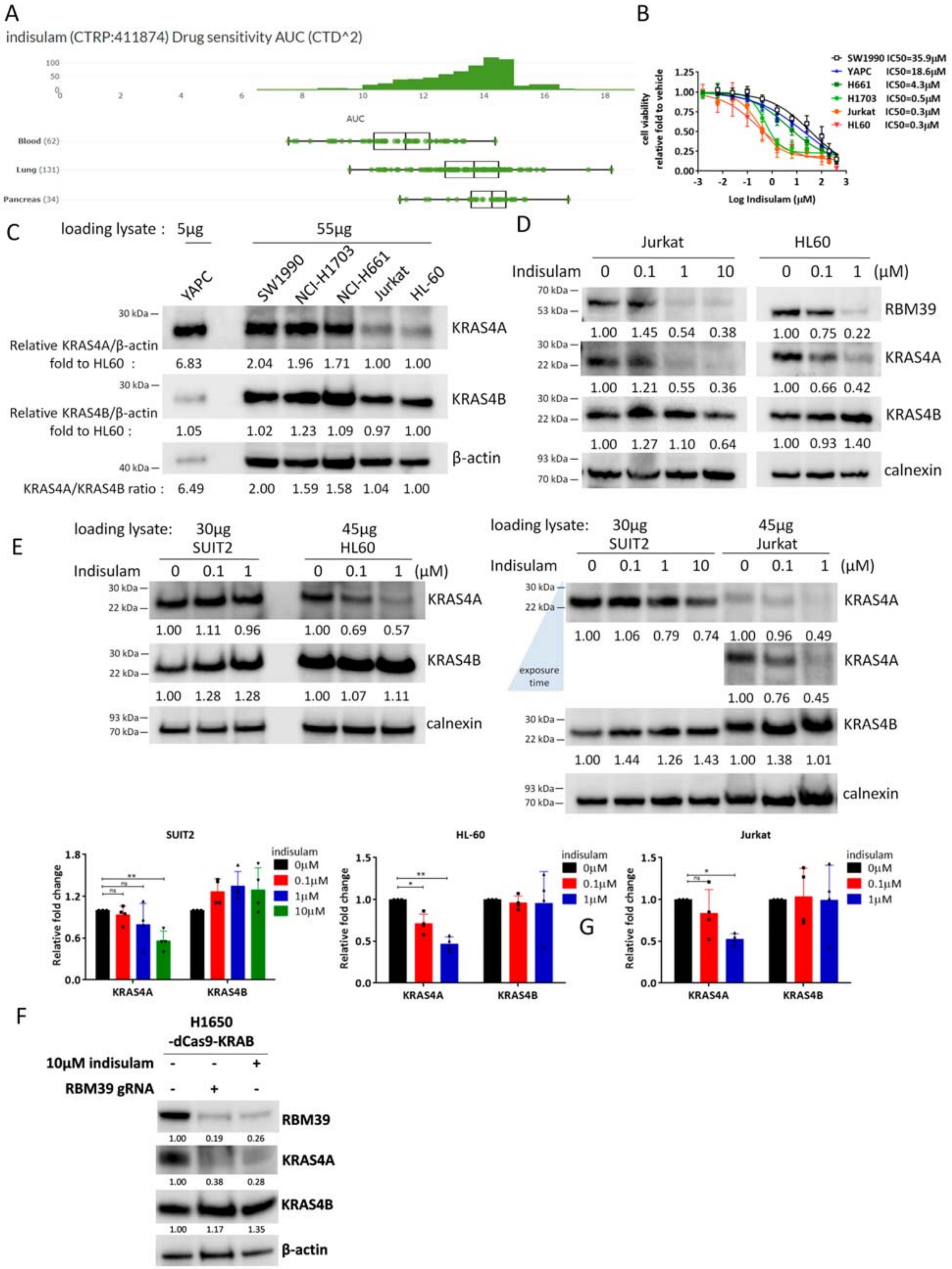
A
indisulam (CTRP:411874) Drug sensitivity AUC (CTD^2)
AUC
Blood (62)
Lung (131)
Pancreas (34)
B
SW1990 IC50=35.9μM
YAPC IC50=18.6μM
H661 IC50=4.3μM
H1703 IC50=0.5μM
Jurkat IC50=0.3μM
HL60 IC50=0.3μM
cell viability relative fold to vehicle
Log Indisulam (μM)
C
loading lysate : 5μg
55μg
YAPC
SW1990
NCI-H1703
NCI-H661
Jurkat
HL-60
KRAS4A
Relative KRAS4A/β-actin fold to HL60 :
6.83 2.04 1.96 1.71 1.00 1.00
KRAS4B
Relative KRAS4B/β-actin fold to HL60 :
1.05 1.02 1.23 1.09 0.97 1.00
β-actin
KRAS4A/KRAS4B ratio : 6.49 2.00 1.59 1.58 1.04 1.00
D
Jurkat
HL60
Indisulam 0 0.1 1 10 0 0.1 1 (μM)
RBM39
1.00 1.45 0.54 0.38 1.00 0.75 0.22
KRAS4A
1.00 1.21 0.55 0.36 1.00 0.66 0.42
KRAS4B
1.00 1.27 1.10 0.64 1.00 0.93 1.40
calnexin
E
loading lysate: 30μg SUIT2
45μg HL60
Indisulam 0 0.1 1 0 0.1 1 (μM)
KRAS4A
1.00 1.11 0.96 1.00 0.69 0.57
KRAS4B
1.00 1.28 1.28 1.00 1.07 1.11
calnexin
loading lysate: 30μg SUIT2
45μg Jurkat
Indisulam 0 0.1 1 10 0 0.1 1 (μM)
KRAS4A
1.00 1.06 0.79 0.74 1.00 0.96 0.49
exposure time
KRAS4A
1.00 0.76 0.45
KRAS4B
1.00 1.44 1.26 1.43 1.00 1.38 1.01
calnexin
SUIT2
HL-60
Jurkat
G
indisulam
0μM
0.1μM
1μM
10μM
Relative fold change
KRAS4A
KRAS4B
F
H1650 -dCas9-KRAB
10μM indisulam - - +
RBM39 gRNA - + -
RBM39
1.00 0.19 0.26
KRAS4A
1.00 0.38 0.28
KRAS4B
1.00 1.17 1.35
β-actin

Figure S7. Effect of Indisulam on *KRAS4A* expression across blood, lung and pancreas cancer cell lines. (A) DEPMAP analysis of sensitivity to indisulam for blood (n=62), lung (n=131) and pancreas cancer cell lines (n=34). The center line is the median, the bottom of the box is the 25th percentile boundary, the top of the box the 75th, and the whiskers define the bounds of the data. (B) The effects of indisulam on growth of a range of cancer cell types from blood, lung and pancreas, showing the strongest inhibition in HL60 and Jurkat cells. Data are presented as mean ± s.d from n=3 independent experiments in HL60, H661, and H1703 cells, and n=4 independent experiments in Jurkat, YAPC and SW1990 cells. (C) Western blot of KRAS4A and KRAS4B for blood, lung and pancreas cancer cell lines. (D) The effect of indisulam on KRAS4A and RBM39 levels in leukemia cell lines. (E) The effects of indisulam on KRAS4A and KRAS4B levels in leukemia and pancreas cancer cell lines. Lower panels show the quantification using ImageJ of KRAS4A and KRAS4B levels after indisulam treatment. Data are presented as mean ± s.d from n=4 independent experiment. *$P < 0.05$; **$P < 0.01$ by two-way ANOVA with Dunnetts's multiple comparison. (F)The effect of indisulam on KRAS4A levels in WT KRAS and RBM39 knockdown human H1650 lung cancer cells.

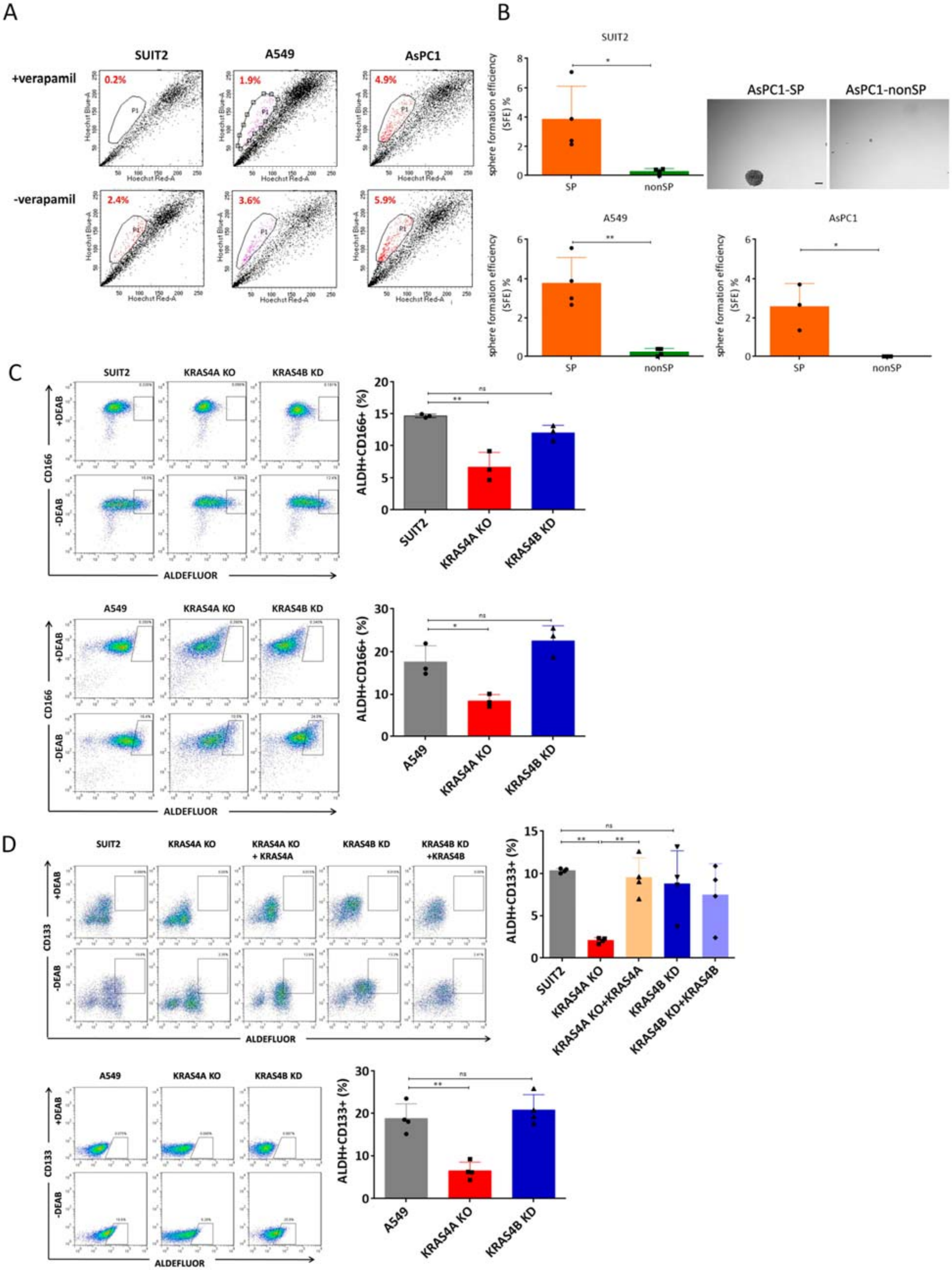
A
SUIT2
A549
AsPC1
+verapamil
-verapamil
0.2%
1.9%
4.9%
2.4%
3.6%
5.9%
Hoechst Blue-A
Hoechst Red-A
P1
B
SUIT2
A549
AsPC1
sphere formation efficiency (SFE) %
SP
nonSP
AsPC1-SP
AsPC1-nonSP
C
SUIT2
KRAS4A KO
KRAS4B KD
A549
+DEAB
-DEAB
CD166
ALDEFLUOR
ALDH+CD166+ (%)
ns
D
KRAS4A KO + KRAS4A
KRAS4B KD +KRAS4B
KRAS4A KO+KRAS4A
KRAS4B KD+KRAS4B
CD133
ALDH+CD133+ (%)

Figure S8. *KRAS4A* regulates stemness properties of cancer cells. (A) Gating strategy for purification of side population cells. Side population cells were identified in SUIT2, A549 and AsPC1 following Hoechst 33342 staining in the presence or absence of verapamil by FACS analysis using blue and red-wavelength assessment. (B) Graph quantifying the sphere formation efficiency in ultra-low attachment plate of side population cells derived from SUIT2, A549 and AsPC1 cell lines. Representative images of spheres derived from AsPC1 cells. Data are presented as mean ± s.d from n=4 independent experiments in SUIT2 and A549 cells, and n=3 independent experiments in AsPC1 cells. *$P < 0.05$; **$P < 0.01$ by unpaired two-tailed *t*-test. The scale bar is presented as 100 μm. (C-D) Loss of KRAS4A reduces the proportion of ALDH+CD166+ (C) and ALDH+CD133+ (D) in SUIT2 and A549 cells. Data are presented as mean ± s.d from n=3 independent experiment in (C) and n=4 independent experiment in (D). *$P < 0.05$; **$P < 0.01$ by two-way ANOVA with Dunnetts's multiple comparison.

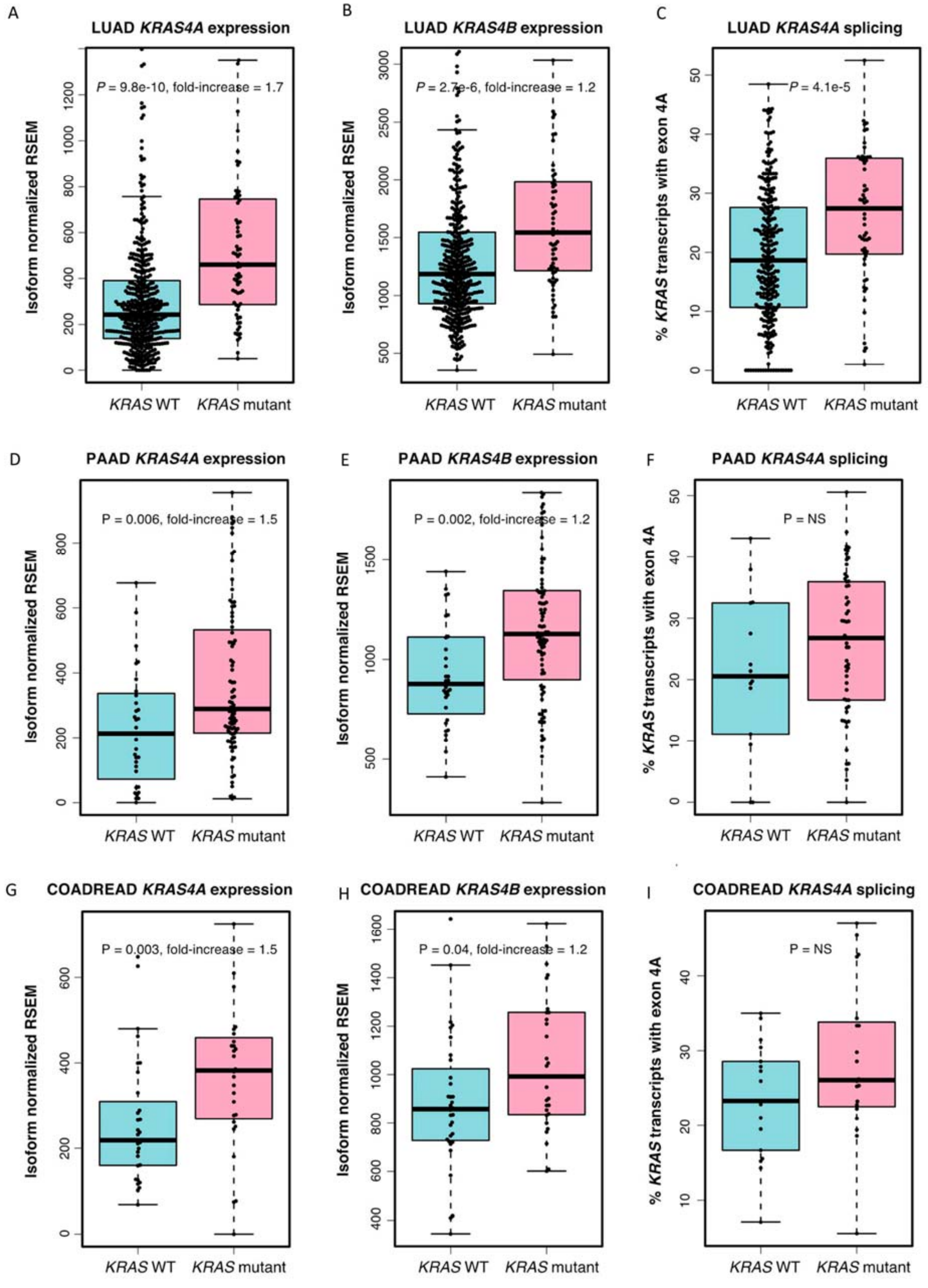

Figure S9. *KRAS4A* and *KRAS4B* expression in cancer. The expression levels of *KARS4A* and

KRAS4B, and the RNA seq reads of KRAS4A transcripts were assessed in lung adenocarcinoma (A-C) (n=422 for *KRAS4A* wildtype and n=68 for *KRAS* mutant lung tumors), pancreatic adenocarcinoma (D-F) (n=322 for *KRAS4A* wildtype and n=888 for *KRAS* mutant pancreatic cancer tumors) and colorectal adenocarcinoma (G-I) (n=32 for *KRAS4A* wildtype and n=28 for *KRAS* mutant lung tumors) were assessed from TCGA datasets. Data are presented as mean ± s.d. *P* value were calculated by unpaired two-tailed *t*-test. The sample sizes were listed in the source data. The center line is the median, the bottom of the box is the 25th percentile boundary, the top of the box the 75th, and the whiskers define the bounds of the data that are not considered outliers, with outliers defined as greater/lesser than +/- 1.5 x IQR, where IQR = inter quartile range.

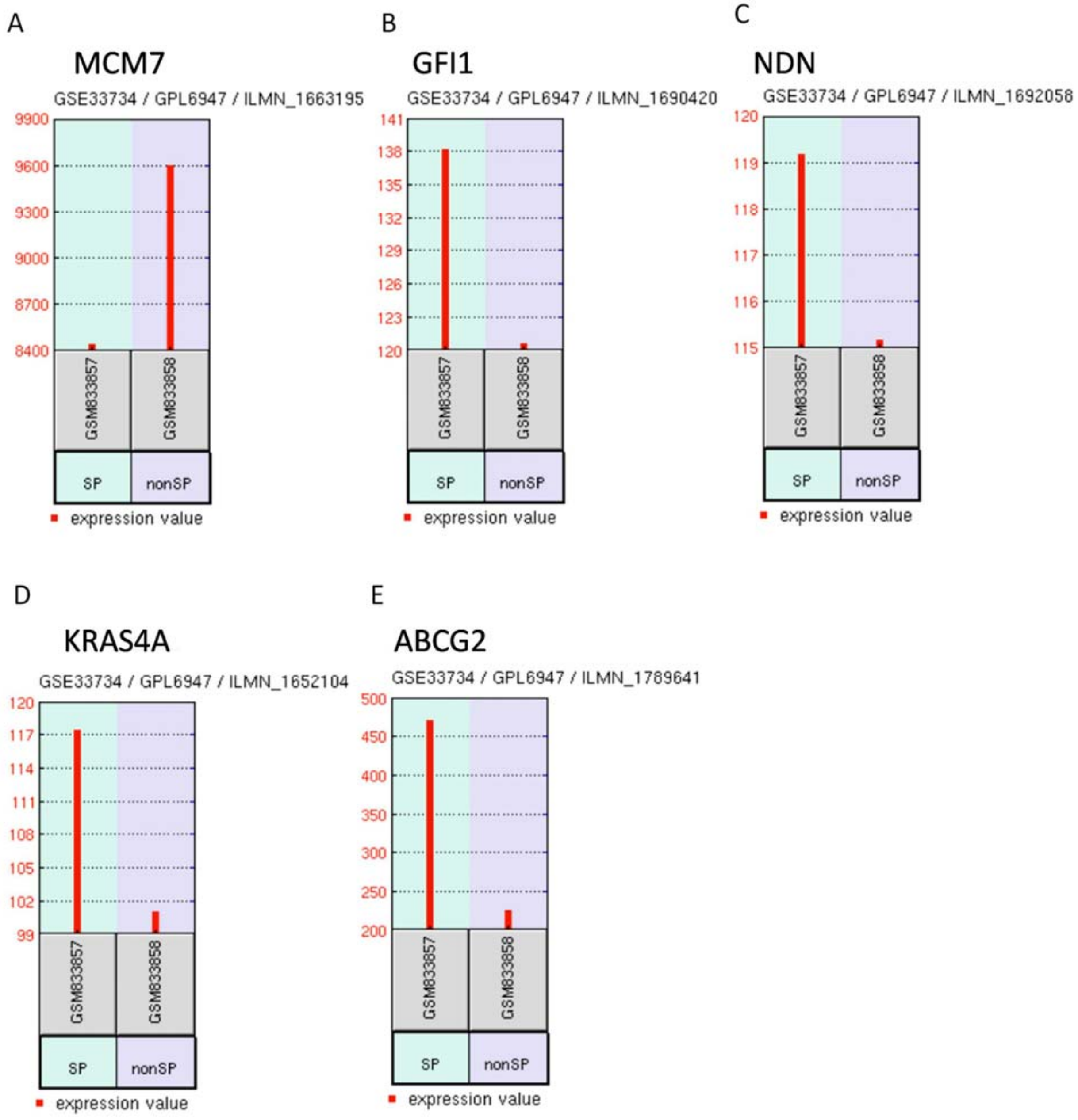

Figure S10. Expression of KRAS4A, ABCG2, MCM7, GFI1 and NDN in side population and non-side population cells from GSE33734 dataset.

Supplementary Table 1: *ras* mutation in carcinogen induced lung tumors

	kras Q61R	*kras* Q61L	*kras* Q61H	*kras* G12D
5 doses of urethane	8/13 (61.5%)	4/13 (30.8%)	-	-
3 doses of urethane	15/36 (41.7%)	12/36 (33.3%)	6/36 (16.7%)	1/36 (2.8%)
3 doses of MNU	1/63 (1.6%)	-	1/63 (1.6%)	53/63 (84.1%)

Supplementary Table 2. Top 100 different genes between high and low KRAS4A/KRAS4B group

MTHFD1	NCAPG2	GREM1	MRPL55
TPX2	ASPM	DSN1	POLE2
MCM6	RRM2	HIST1H2BC	RFC4
CDKN3	MCM2	DTL	RAN
KNTC1	PSMD2	MKI67	HIST1H4L
KIAA1524	HIST1H2AG	SERPINH1	TK1
SPATS2	HIST1H1B	CHEK1	CEP55
FAP	DNMT1	CCT4	PLK4
TMEM194A	RPSA	VCAN	THOC4
BRIP1	MYBL2	CKAP2L	RFC2
LMNB1	CDCA4	RPL36	RNF34
VHL	MPHOSPH9	KIFC1	RPL29
PDPN	RACGAP1	DLGAP5	WDR5
NCAPG	RPSA	SNORD30	PRKDC
MARS	WDHD1	UBE2T	GSTO1
FANCD2	BUB1	COL1A2	WISP1
MCM7	NCAPH	NUSAP1	FAM72D
LIG1	IQGAP3	HIST2H2BE	INCENP
TACC3	HIST1H3B	CCT2	MCM3
KIF20B	BCAT1	BUB1B	NME2P1
POLA2	PRR11	TTL	THOC4
NEU3	MELK	YBX1P2	TUBA1B
TRMT1	TOP2A	ADA	CCNE2
KIFC1	NCAPD3	INHBA	KIF11
RNASEH2A	CENPF	IMPDH2	NUP37

Mutant KRAS LUAD samples were obtained from UCSF and the samples are split into high and low group based on KRAS4A/KRAS4B ratio (High group: upper KRAS4A/KRAS4B ratio sample, n=8; Low group: lower KRAS4A/KRAS4B ratio sample, n=7). The significantly expressed gene between high and low group was demonstrated by CARMEN.

Supplementary Table 3. GO biological process in LUAD samples

GO biological process complete	raw P-value	FDR
negative regulation of DNA duplex unwinding (GO:1905463)	1.95E-04	2.31E-02
pre-replicative complex assembly involved in cell cycle DNA replication (GO:1902299)	1.19E-07	3.33E-05
pre-replicative complex assembly involved in nuclear cell cycle DNA replication (GO:0006267)	1.19E-07	3.27E-05
pre-replicative complex assembly (GO:0036388)	1.19E-07	3.21E-05
nuclear cell cycle DNA replication initiation (GO:1902315)	2.91E-04	3.20E-02
cell cycle DNA replication initiation (GO:1902292)	2.91E-04	3.18E-02
mitotic DNA replication initiation (GO:1902975)	2.91E-04	3.15E-02
RNA localization to nucleus (GO:0090685)	4.07E-04	4.15E-02
RNA localization to Cajal body (GO:0090670)	4.07E-04	4.12E-02
scaRNA localization to Cajal body (GO:0090666)	4.07E-04	4.10E-02
meiotic sister chromatid cohesion, centromeric (GO:0051754)	4.07E-04	4.07E-02
regulation of DNA duplex unwinding (GO:1905462)	4.07E-04	4.04E-02
double-strand break repair via break-induced replication (GO:0000727)	4.86E-07	1.12E-04
mitotic DNA replication (GO:1902969)	4.86E-07	1.11E-04
mitotic chromosome condensation (GO:0007076)	2.09E-06	4.26E-04
DNA strand elongation involved in DNA replication (GO:0006271)	2.09E-06	4.21E-04
DNA unwinding involved in DNA replication (GO:0006268)	5.57E-05	7.78E-03
nuclear DNA replication (GO:0033260)	1.77E-12	1.41E-09
prophase (GO:0051324)	6.67E-05	8.91E-03
mitotic prophase (GO:0000088)	6.67E-05	8.84E-03
cell cycle DNA replication (GO:0044786)	2.11E-12	1.60E-09

DNA replication initiation (GO:0006270)	1.64E-08	5.93E-06
DNA strand elongation (GO:0022616)	5.13E-06	9.60E-04
chromosome condensation (GO:0030261)	2.38E-09	1.05E-06
telomere maintenance via semi-conservative replication (GO:0032201)	8.07E-06	1.44E-03
error-prone translesion synthesis (GO:0042276)	1.42E-04	1.76E-02
nucleotide-excision repair, DNA gap filling (GO:0006297)	2.07E-04	2.42E-02
mitotic spindle assembly checkpoint (GO:0007094)	2.88E-04	3.25E-02
mitotic spindle checkpoint (GO:0071174)	2.88E-04	3.23E-02
spindle assembly checkpoint (GO:0071173)	2.88E-04	3.20E-02
spindle checkpoint (GO:0031577)	2.88E-04	3.18E-02
mitotic spindle assembly (GO:0090307)	2.99E-05	4.49E-03
negative regulation of mitotic metaphase/anaphase transition (GO:0045841)	3.52E-04	3.76E-02
negative regulation of metaphase/anaphase transition of cell cycle (GO:1902100)	3.87E-04	4.00E-02
G1/S transition of mitotic cell cycle (GO:0000082)	7.35E-13	6.50E-10
cell cycle G1/S phase transition (GO:0044843)	8.05E-13	6.74E-10
translesion synthesis (GO:0019985)	4.79E-05	6.93E-03
negative regulation of mitotic sister chromatid separation (GO:2000816)	4.64E-04	4.56E-02
chromosome separation (GO:0051304)	4.64E-04	4.53E-02
mitotic sister chromatid segregation (GO:0000070)	9.61E-11	5.66E-08
sister chromatid segregation (GO:0000819)	1.41E-13	1.72E-10
negative regulation of chromosome separation (GO:1905819)	5.06E-04	4.91E-02
DNA-dependent DNA replication (GO:0006261)	1.42E-11	9.42E-09
negative regulation of mitotic nuclear division (GO:0045839)	6.20E-05	8.42E-03

chromosome organization involved in meiotic cell cycle (GO:0070192)	8.22E-07	1.82E-04
mitotic nuclear division (GO:0140014)	3.93E-12	2.84E-09
regulation of mitotic sister chromatid separation (GO:0010965)	1.14E-05	1.87E-03
mitotic spindle organization (GO:0007052)	1.54E-06	3.22E-04
DNA synthesis involved in DNA repair (GO:0000731)	1.06E-04	1.38E-02
DNA replication (GO:0006260)	4.16E-15	8.28E-12
regulation of chromosome separation (GO:1905818)	1.63E-05	2.57E-03
negative regulation of nuclear division (GO:0051784)	1.22E-04	1.57E-02
postreplication repair (GO:0006301)	1.22E-04	1.55E-02
regulation of chromosome segregation (GO:0051983)	4.60E-08	1.38E-05
nuclear chromosome segregation (GO:0098813)	5.48E-15	8.72E-12
positive regulation of DNA biosynthetic process (GO:2000573)	1.87E-05	2.91E-03
DNA biosynthetic process (GO:0071897)	5.63E-08	1.66E-05
regulation of mitotic metaphase/anaphase transition (GO:0030071)	1.40E-04	1.75E-02
regulation of metaphase/anaphase transition of cell cycle (GO:1902099)	1.59E-04	1.95E-02
regulation of mitotic sister chromatid segregation (GO:0033047)	2.42E-05	3.67E-03
microtubule cytoskeleton organization involved in mitosis (GO:1902850)	6.10E-07	1.37E-04
meiotic chromosome segregation (GO:0045132)	4.48E-06	8.48E-04
nuclear division (GO:0000280)	9.75E-16	2.22E-12
DNA duplex unwinding (GO:0032508)	8.75E-07	1.91E-04
DNA packaging (GO:0006323)	7.64E-10	3.92E-07
telomere maintenance (GO:0000723)	7.12E-06	1.30E-03
mitotic cell cycle process (GO:1903047)	1.65E-30	8.76E-27

regulation of sister chromatid segregation (GO:0033045)	4.88E-05	6.99E-03
telomere organization (GO:0032200)	8.39E-06	1.48E-03
DNA geometric change (GO:0032392)	1.45E-06	3.07E-04
chromosome segregation (GO:0007059)	1.53E-13	1.74E-10
organelle fission (GO:0048285)	4.45E-15	7.86E-12
mitotic cell cycle phase transition (GO:0044772)	1.70E-13	1.80E-10
spindle assembly (GO:0051225)	5.15E-05	7.25E-03
M phase (GO:0000279)	7.77E-09	3.25E-06
mitotic M phase (GO:0000087)	7.77E-09	3.17E-06
regulation of mitotic nuclear division (GO:0007088)	8.19E-09	3.18E-06
DNA conformation change (GO:0071103)	3.60E-14	4.78E-11
cell cycle phase transition (GO:0044770)	2.60E-13	2.43E-10
mitotic cytokinesis (GO:0000281)	3.53E-04	3.74E-02
mitotic cell cycle (GO:0000278)	8.26E-30	3.28E-26
regulation of nuclear division (GO:0051783)	2.81E-08	9.53E-06
negative regulation of chromosome organization (GO:2001251)	4.08E-06	7.92E-04
spindle organization (GO:0007051)	4.47E-06	8.56E-04
meiotic cell cycle process (GO:1903046)	1.11E-06	2.39E-04
cell division (GO:0051301)	5.72E-17	1.52E-13
meiotic nuclear division (GO:0140013)	7.16E-06	1.30E-03
cell cycle checkpoint (GO:0000075)	3.67E-07	9.13E-05
regulation of DNA biosynthetic process (GO:2000278)	1.72E-04	2.08E-02
protein-DNA complex assembly (GO:0065004)	3.98E-07	9.46E-05

cell cycle process (GO:0022402)	8.03E-33	1.28E-28
anaphase (GO:0051322)	8.43E-06	1.47E-03
mitotic anaphase (GO:0000090)	8.43E-06	1.46E-03

Mutant KRAS LUAD samples were obtained from UCSF and GO biological process was demonstrated by Gene ontology (GO) enrichment analysis based on the significantly expressed gene between high and low group in supplementary Table 2

Supplementary Table 4. Enrichment pathway in low KRAS4A/KRAS4B ratio LUAD samples

Gene set name	ES	NES	NOM p-val	FDR q-val
REACTOME_CELL_CYCLE	0.5322	1.7114	0.0290	0.1894
REACTOME_DNA_REPAIR	0.5423	1.6892	0.0355	0.1657
REACTOME_NUCLEOTIDE_EXCISION_REPAIR	0.5278	1.6785	0.0334	0.0334
REACTOME_CELL_CYCLE_CHECKPOINTS	0.5807	1.6503	0.0268	0.1655
REACTOME_FORMATION_OF_TRANSCRIPTION_COUPLED_NER_TC_NER_REPAIR_COMPLEX	0.5600	1.6468	0.0328	0.1647
REACTOME_INHIBITION_OF_THE_PROTTEOLYTIC_ACTIVITY_OF_APC_C_REQUIRED_FOR_THE _ONSET_OF_ANAPHASE_BY_MITOTIC_SPINDLE_CHECKPOINT_COMPONENTS	0.6528	1.6239	0.0345	0.1713
REACTOME_PHOSPHORYLATION_OF_THE_APC_C	0.6478	1.6042	0.0311	0.1716
REACTOME_APC_C_CDC20_MEDIATED_DEGRADATION_OF_MITOTIC_PROTEINS	0.5403	1.5985	0.0494	0.1687
REACTOME_CONVERSION_FROM_APC_C_CDC20_TO_APC_C_CDH1_IN_LATE_ANAPHASE	0.5508	1.5937	0.0361	0.1583
REACTOME_APC_C_CDC20_MEDIATED_DEGRADATION_OF_CYCLIN_B	0.6273	1.5896	0.0363	0.1555
REACTOME_CHROMOSOME_MAINTENANCE	0.4990	1.5301	0.0373	0.1496
REACTOME_ACTIVATION_OF_ATR_IN_RESPONSE_TO_REPLICATION_STRESS	0.7381	1.5296	0.0297	0.1479
REACTOME_G0_AND_EARLY_G1	0.7141	1.5247	0.0450	0.1509
REACTOME_G2_M_CHECKPOINTS	0.7318	1.5163	0.0487	0.1541
REACTOME_ACTIVATION_OF_THE_PRE_REPLICATIVE_COMPLEX	0.7585	1.5020	0.0414	0.1626

Mutant KRAS LUAD samples were obtained from TCGA and the samples are split into high and low groups based on KRAS4A/KRAS4B ratio

(High group: 10% upper KRAS4A/KRAS4B ratio sample, n=7; Low group: 10% lower KRAS4A/KRAS4B ratio sample, n=7). Enrichment pathway was demonstrated by gene set enrichment analysis (GSEA) using the REACTOME pathway gene sets.

Supplementary Table.5. List of primers	
Sequencing primer for KRAS4A-Forward	caaaccaggattctagcccata
Sequencing primer for KRAS4A-Reversed	gtggttgccaccttgttacc
Sequencing primer for KRAS4B-Forward	ttcagttgcctgaagagaaaca
Sequencing primer for KRAS4B-Reversed	agtctgcatggagcaggaaa
KRA4A gRNA-Top	CACCGGGAGGATGCTTTTTATACAT
KRA4A gRNA-Bottom	AAACATGTATAAAAAGCATCCTCCC
KRA4B gRNA-Top	CACCGTTCTCGAACTAATGTATAGA
KRA4B gRNA- Bottom	AAACTCTATACATTAGTTCGAGAAC
RBM39 gRNA.1-Top	TTGAGCAGCGGCCGCCATTTCAGTTTAAGAGC
RBM39 gRNA.1-Bottom	TTAGCTCTTAAACTGAAATGGCGGCCGCTGCTCAACAAG
RBM39 gRNA.2-Top	TTGGAGAGCAGGACGGCGGCTTGTTTAAGAGC
RBM39 gRNA.2-Bottom	TTAGCTCTTAAACAAGCCGCCGTCCTGCTCTCCAACAAG
CA9 gRNA.1-Top	TTGGGATCAACAGAGGGAGCCA GTTTAAGAGC
CA9 gRNA.1-Bottom	TTAGCTCTTAAACtggctccctctgttgatcccAACAAG
CA9 gRNA.2-Top	TTGCAGGGGCCGGGATCAACAGGTTTAAGAGC
CA9 gRNA.2-Bottom	TTAGCTCTTAAAC ctgttgatcccggcccctgc AACAAG
Taqman-KRAS4A-F	TGTGATTTGCCTTCTAGAACAGTAGAC
Taqman-KRAS4A-R	CTCACCAATGTATAAAAAGCATCCTC
Taqman-KRAS4B-F	TGTGATTTGCCTTCTAGAACAGTAGAC
Taqman-KRAS4B-R	GGCATCATCAACACCCTGTC
