# Supplementary material for: Targeting KRAS4A splicing through the RBM39/DCAF15 pathway inhibits cancer stem cells
Source: Nat Commun. 2021 Jul 13;12:4288. doi: 10.1038/s41467-021-24498-7 (PMC8277813; doi:10.1038/s41467-021-24498-7)
Supplement: Supplementary file 3 — Reporting Summary [file 41467_2021_24498_MOESM3_ESM.pdf]

nature research

Corresponding author(s): Allan Balmain

Last updated by author(s): 2021/4/29

Reporting Summary

Nature Research wishes to improve the reproducibility of the work that we publish. This form provides structure for consistency and transparency in reporting. For further information on Nature Research policies, see our Editorial Policies and the Editorial Policy Checklist.

Statistics

For all statistical analyses, confirm that the following items are present in the figure legend, table legend, main text, or Methods section.

n/a	Confirmed	
☐	☒	The exact sample size (*n*) for each experimental group/condition, given as a discrete number and unit of measurement
☐	☒	A statement on whether measurements were taken from distinct samples or whether the same sample was measured repeatedly
☐	☒	The statistical test(s) used AND whether they are one- or two-sided<br>*Only common tests should be described solely by name; describe more complex techniques in the Methods section.*
☒	☐	A description of all covariates tested
☐	☒	A description of any assumptions or corrections, such as tests of normality and adjustment for multiple comparisons
☐	☒	A full description of the statistical parameters including central tendency (e.g. means) or other basic estimates (e.g. regression coefficient) AND variation (e.g. standard deviation) or associated estimates of uncertainty (e.g. confidence intervals)
☐	☒	For null hypothesis testing, the test statistic (e.g. *F*, *t*, *r*) with confidence intervals, effect sizes, degrees of freedom and *P* value noted<br>*Give P values as exact values whenever suitable.*
☒	☐	For Bayesian analysis, information on the choice of priors and Markov chain Monte Carlo settings
☒	☐	For hierarchical and complex designs, identification of the appropriate level for tests and full reporting of outcomes
☐	☐	Estimates of effect sizes (e.g. Cohen's *d*, Pearson's *r*), indicating how they were calculated

Our web collection on statistics for biologists contains articles on many of the points above.

Software and code

Policy information about availability of computer code

Data collection	BD FACSDiva software version 8.0.2 was used for sorting. LI-COR image studio 5.0 for Odyssey Fc was used for WB blot image acquisition. QuantStudio 5 real-time PCR system software was used for Taqman assay.
Data analysis	The isoform-specific expression analyses were analyzed and non-parametric statistical tests performed in R-3.4.0. Plots were generated using the R package ggplot2 (H. Wickham. ggplot2: Elegant Graphics for Data Analysis. Springer-Verlag New York, 2009.) Gene Set Enrichment Analysis (GSEA) was performed using curated gene sets (c2.cp.reactome.v6.2.symbol.bmt). The colony area in soft agar growth assay was quantified using Image-Pro Plus 5. BD FACSDiva software version 8.0.2 was used for data analysis. GraphPad Prism 6.01 was used for statistical test analysis.

For manuscripts utilizing custom algorithms or software that are central to the research but not yet described in published literature, software must be made available to editors and reviewers. We strongly encourage code deposition in a community repository (e.g. GitHub). See the Nature Research guidelines for submitting code & software for further information.

Data

Policy information about availability of data

All manuscripts must include a data availability statement. This statement should provide the following information, where applicable:

- Accession codes, unique identifiers, or web links for publicly available datasets
- A list of figures that have associated raw data
- A description of any restrictions on data availability

The authors declare that the data supporting the findings of this study are available within the paper and its supplementary information files. The TCGA, CCLE, depmap and microarray data are publicly available datasets. The gene expression microarray data was downloaded from the NCBI Gene Expression Omnibus with accession numbers GSE33734 https://www.ncbi.nlm.nih.gov/geo/query/acc.cgi?acc=GSE33734. The gene expression data from the TCGA project were downloaded

from the UCSC cancer browser http://xena.ucsc.edu/welcome-to-ucsc-xena/. The drug sensitivity was downloaded from depmap portal https://depmap.org/portal/. The gene expression was downloaded from CCLE https://portals.broadinstitute.org/ccle. All relevant data are available from the authors. Source data are provided with this paper. The remaining data are available within the Article, Supplementary Information or available from the authors upon request

Field-specific reporting

Please select the one below that is the best fit for your research. If you are not sure, read the appropriate sections before making your selection.

☒ Life sciences ☐ Behavioural & social sciences ☐ Ecological, evolutionary & environmental sciences

For a reference copy of the document with all sections, see nature.com/documents/nr-reporting-summary-flat.pdf

Life sciences study design

All studies must disclose on these points even when the disclosure is negative.

Sample size	Animal numbers were based on extensive previous studies (PMID:18758463) of carcinogenesis using mouse models and are described in Methods and/or Figure legends
Data exclusions	no data exclusions
Replication	All biological experiments were repeated using different cell lines, and at least 3 replicate studies were carried out as described in the figure legends.
Randomization	Tumor xenografts were evaluated for the effect of inhibitors on tumor growth. Mice (n=10) with tumor size of 50~100mm3 were randomized into groups for vehicle and inhibitor treatment.
Blinding	Blinding was not applicable to animal experiment as animal are assigned to specific treatment.

Reporting for specific materials, systems and methods

We require information from authors about some types of materials, experimental systems and methods used in many studies. Here, indicate whether each material, system or method listed is relevant to your study. If you are not sure if a list item applies to your research, read the appropriate section before selecting a response.

Materials & experimental systems

n/a	Involved in the study	
☐	☒	Antibodies
☐	☒	Eukaryotic cell lines
☒	☐	Palaeontology and archaeology
☐	☒	Animals and other organisms
☒	☐	Human research participants
☒	☐	Clinical data
☒	☐	Dual use research of concern

Methods

n/a	Involved in the study	
☒	☐	ChIP-seq
☐	☒	Flow cytometry
☒	☐	MRI-based neuroimaging

Antibodies

Antibodies used	The antibodies used were KRAS4A (clone 10C11E4; custom antibody), KRAS4B (clone 3B10-2F2; WH0009584M1; Sigma-Aldrich), β-actin (clone C4; sc47778; Santa-Cruz Biotech), RBM39 (clone 4G8; WH0009584M1; Sigma-Aldrich), Hras (clone C20; sc520; Santa-Cruz Biotech), Nras (clone C20; sc519; Santa-Cruz Biotech), phospho-Akt (clone D9E, cat#4060, Cell Signaling Technology), Phospho-Erk1/2 (clone D13.14.4E, cat#4370, Cell Signaling Technology), Phospho-MEK1/2 (clone 166F8, cat#2338, Cell Signaling Technology). The secondary antibodies used were anti-mouse IgG-HRP (cat#7076, Cell Signaling Technology), anti-rabbit IgG-HRP (cat#7074,Cell Signaling Technology) and anti-Rat IgG-HRP (NA935, Millipore-Sigma)
Validation	KRAS4A, KRAS4B, andβ-actin antibodies were valiated by western blotting analysis in human cancer cell lines and MEFs. RBM39 antibody was valiated by western blotting analysis in human cancer cell lines. Hras, Nras, phospho-Akt, Phospho- Erk1/2, Phospho-MEK1/2 were valiated by western blotting analysis in MEFs.

Eukaryotic cell lines

Policy information about cell lines

Cell line source(s)	Human cell lines SUIT2 was from AcceGen. A549, H358 and AsPC1 were from ATCC.
Authentication	All human human cell lines were obtained from commercial suppliers with authentication by STR profiling

Mycoplasma contamination	The cell lines were tested negative for mycoplasma contamination using PCR Mycoplasma Detection Kit (cat# G238, Applied Biological Materials Inc (abm).
Commonly misidentified lines (See ICLAC register)	no commonly misidentified cell lines were used in this study

Animals and other organisms

Policy information about studies involving animals; ARRIVE guidelines recommended for reporting animal research

Laboratory animals	FVB/N strains male and female mice at 8-10 weeks of age for lung tumorigenesis study. Nude male mice and NSG male mice at 6 weeks of age for xenograft model. The housing conditions for the mice is 12 light/12 dark cycle with temperatures of 68-79 degrees F and 30-70% humidity.
Wild animals	The study did not involve wild animals
Field-collected samples	The study did not involve samples collected from the field
Ethics oversight	The protocol was approved by the UCSF IUCAC (AN102384-03D)

Note that full information on the approval of the study protocol must also be provided in the manuscript.

Flow Cytometry

Plots

Confirm that:

- [x] The axis labels state the marker and fluorochrome used (e.g. CD4-FITC).
- [x] The axis scales are clearly visible. Include numbers along axes only for bottom left plot of group (a 'group' is an analysis of identical markers).
- [x] All plots are contour plots with outliers or pseudocolor plots.
- [x] A numerical value for number of cells or percentage (with statistics) is provided.

Methodology

Sample preparation	The samples include lung cancer cell A549 and pancreatic cancer cell SUIT2 and AsPC1. For side population analysis, cells were suspended at one million cells per ml in DMEM containing 2% FBS and 10mM HEPES.
Instrument	BD FACSAria flow cytometry was used in this study.
Software	BD FACSDiva™ software was used to collect and analyze the flow cytometry data
Cell population abundance	The ABCG2 mRNA was determined by qPCR assay within post-sort fraction of side population.
Gating strategy	Side population was gated on the flow cytometric profile based on Hoechst red and Hoechst blue channel displayed in the linear scale, and ABC transporter inhibitor verapamil was used to define the boundary between the SP and non-SP cell population.

- [x] Tick this box to confirm that a figure exemplifying the gating strategy is provided in the Supplementary Information.
